# Multiscale X-ray phase contrast imaging of human cartilage for investigating osteoarthritis formation

**DOI:** 10.1186/s12929-021-00739-1

**Published:** 2021-06-07

**Authors:** Annie Horng, Johannes Stroebel, Tobias Geith, Stefan Milz, Alexandra Pacureanu, Yang Yang, Peter Cloetens, Goran Lovric, Alberto Mittone, Alberto Bravin, Paola Coan

**Affiliations:** 1 Department of Clinical Radiology, Faculty of Medicine, Ludwig-Maximilians-University, Marchioninistr. 15, 81377 Munich, Germany; 2 RZM – Radiologisches Zentrum Munich-Pasing, Pippinger Str. 25, 81245 Munich, Germany; 3 Department of Medical Physics, Faculty of Physics, Ludwig-Maximilians-University Munich, Am Coulombwall 1, 85748 Garching, Germany; 4 Department of Interventional Radiology, Klinikum Rechts der Isar of the Technical University of Munich, Munich, Germany; 5 Faculty of Medicine, Anatomische Anstalt, Neuroanatomy, Ludwig Maximilians University, Munich, Germany; 6 European Synchrotron Radiation Facility, Grenoble, France; 7 National Synchrotron Light Source II, Brookhaven National Laboratory, Upton, NY 11973 USA; 8 Paul Scherrer Institute (Swiss Light Source), Villigen, Switzerland; 9 CELLS: ALBA Synchrotron, Barcelona, Spain

**Keywords:** X-ray phase-contrast imaging, Cartilage, Osteoarthritis, 3D analysis, Virtual histology

## Abstract

**Background:**

The evolution of cartilage degeneration is still not fully understood, partly due to its thinness, low radio-opacity and therefore lack of adequately resolving imaging techniques. X-ray phase-contrast imaging (X-PCI) offers increased sensitivity with respect to standard radiography and CT allowing an enhanced visibility of adjoining, low density structures with an almost histological image resolution. This study examined the feasibility of X-PCI for high-resolution (sub-) micrometer analysis of different stages in tissue degeneration of human cartilage samples and compare it to histology and transmission electron microscopy.

**Methods:**

Ten 10%-formalin preserved healthy and moderately degenerated osteochondral samples, post-mortem extracted from human knee joints, were examined using four different X-PCI tomographic set-ups using synchrotron radiation the European Synchrotron Radiation Facility (France) and the Swiss Light Source (Switzerland). Volumetric datasets were acquired with voxel sizes between 0.7 × 0.7 × 0.7 and 0.1 × 0.1 × 0.1 µm^3^. Data were reconstructed by a filtered back-projection algorithm, post-processed by ImageJ, the WEKA machine learning pixel classification tool and VGStudio max. For correlation, osteochondral samples were processed for histology and transmission electron microscopy.

**Results:**

X-PCI provides a three-dimensional visualization of healthy and moderately degenerated cartilage samples down to a (sub-)cellular level with good correlation to histologic and transmission electron microscopy images. X-PCI is able to resolve the three layers and the architectural organization of cartilage including changes in chondrocyte cell morphology, chondrocyte subgroup distribution and (re-)organization as well as its subtle matrix structures.

**Conclusions:**

X-PCI captures comprehensive cartilage tissue transformation in its environment and might serve as a tissue-preserving, staining-free and volumetric virtual histology tool for examining and chronicling cartilage behavior in basic research/laboratory experiments of cartilage disease evolution.

## Background

Cartilage alterations are considered initiators of osteoarthritis (OA) [1–3]. Cartilage, as a sparsely cell populated and avascular tissue, has only limited reparative potential [4]. The detailed evolution of cartilage breakdown with its consecutive changes of biomechanical properties and alterations in joint mechanics, is still incompletely understood because of the lack of appropriate imaging techniques or biomarkers to monitor this process.

Clinical computed tomography (CT), as well as standard laboratory-based micro-CT set-ups, lack the contrast resolution to sufficiently visualize cartilage composition and its pathological alterations due to poor X-ray absorption of the tissue. The current clinical method of choice for direct cartilage depiction is Magnetic Resonance Imaging (MRI), which provides anatomical information about tissue volume and height [5], structural matrix changes and defects [6] and quantitative biochemical properties such as collagen or proteoglycan content [7–9], but the depiction varies strongly depending on the MRI sequence used [6]. Cautious interpretation is necessary as for a given lesion the estimated size differs depending on the chosen sequence parameters, which signifies that the application of a standardized protocol is very important to achieve meaningful consistent lesion size estimation. Currently, MRI techniques reach spatial resolutions down to 0.4 × 0.4 × 0.4 mm^3^ using isotropic 3D sequences. These resolutions provide proper depiction of medium to advanced cartilage changes, but are only partly sufficient to reveal early tissue disturbances such as softening and superficial fibrillation; also, cellular cartilage components are not resolved. While morphological MRI is limited by satisfactory spatial and contrast resolutions, quantitative MRI techniques for analyzing cartilage matrix composition (collagen/proteoglycan content), such as delayed gadolinium-enhanced MRI of cartilage, sodium imaging [10] or T2 relaxation time mapping [11], have not been established in routine examinations because of their time-consuming acquisition, post-processing and resultant low resolution map of matrix changes.

Therefore, the development of more sensitive imaging technologies with the goal to improve resolution and depiction of structural cartilage change is still desirable to (1) obtain an improved and accurate diagnosis of early potentially reversible cartilage change, (2) and foster early initiation of therapy in response to the limited reparative capacity of the tissue. Such new methods could then simultaneously serve as a precise tool for follow-up monitoring during treatment.

X-ray phase contrast imaging (X-PCI) emerged in biomedical diagnostics about twenty years ago with previous reported applications in cartilage research. X-PCI techniques can discriminate regional extracellular matrix and even cell alterations in soft tissues with homogeneous and uniform densities by exploiting both attenuated and refracted X-rays in the traversed tissue [12, 13]. The refraction signal enhances image contrast by orders of magnitude with respect to standard absorption radiology [14–17]. A previous study analyzed a volumetric X-PCI-CT dataset of a fully preserved ex-vivo human knee joint and already confirmed superior depiction and discrimination of soft tissues such as cartilage, ligaments, tendons, capsular structures, muscles and bone in comparison to conventional CT and with similar differentiation of the structures as compared to MRI [17].

Our goal was now to exploit the feasibility of X-PCI to push the limits of high-resolution analysis in cartilage imaging. We applied and implemented state-of-the-art X-PCI setups and image processing methods for imaging of healthy and degenerated cartilage samples in a laboratory setting (1) to study the visibility of the stages in tissue degradation at cellular and sub-cellular levels, and (2) to explore X-PCI image quality in comparison to histology and TEM to evaluate it as a non-invasive virtual histology technique.

## Methods

### Samples

The experiment complied with ethical standards of the ethics committee of the Ludwig-Maximilians-University (Munich, Germany) and with the Helsinki Declaration of 1975, as revised in 2000. We examined 10 cylindrical osteochondral samples (height 15–20 mm, diameter 7 mm) extracted post-mortem from three human knee joints (3 × femoral 82 year-old (yo) female, 3 × patellar 73yo male, 4 × patellar 67 yo female). All specimens were harvested within 24 h of death at the Forensic Medicine department of the Ludwig-Maximilians-University after obtaining informed consent from the donor’s next-of-kin. Samples were preserved in a 10% formalin-saline solution, which reportedly does not influence X-PCI results [15, 18] and prevents degradation during transport and experiment.

Samples were classified as healthy or slight-to-medium degenerated based on macroscopic visual evaluation of the cartilage surface and thickness by an experienced forensic doctor and a musculoskeletal-expert radiologist. A smooth glossy surface was considered an indication of healthy cartilage, slight fibrillations and roughened surface as signs of a medium degenerated cartilage. Advanced degenerated samples were excluded because this study focused on evaluation of early degenerative changes.

Specimens were either placed in cylindrical plastic containers filled with a 10% formalin solution or decalcified, embedded in paraffin blocks and cut into “sticks” with a dimension of 0.5 × 0.5 × 1 mm^3^, depending on the spatial resolution (and thus setup) used for their examination. The plastic containers were then glued on a support screwed on the rotation stage of the micro-/sub-micron-CT setups, whereas the decalcified sticks of cartilage were inserted and glued in specifically hollowed supports clamped to the nano-holotomography set-up.

### Imaging technique

The propagation-based phase-contrast imaging technique was applied to image cartilage samples using both single-distance [19] (for qualitative analysis) and multi-distance nano-holotomography [20] (for quantitative imaging) approaches. While in conventional radiography image contrast is mostly generated by differences in X-ray attenuation in the traversed tissues, the contrast in X-PCI images arises also from X-ray refraction. X-rays are both refracted and absorbed while passing through an object, as described by the index of refraction: $n=1-\delta +i\beta ,$ where $\delta$ is the refraction decrement and $\beta$ the absorption term. The refraction term for soft materials is about 10^3^ times larger than the absorption one in the energy range of radiological interest [21]. By passing through the object, in agreement with Fresnel’s theory, parts of the wavefront can create interference patterns, that generate a modulation of the beam intensity [22] depending on the refraction properties of the sample; this intensity modulation is then recorded by a charged-coupled device (CCD)-based indirect detector. The single distance phase retrieval algorithm, introduced by Paganin et al. which uses assumptions about the material to calculate the phase shift, has been used to produce qualitative phase images of tissue [19]. In order to enhance the contrast and spatial resolutions, quantitative phase contrast images have been obtained by nano-holotomography, a high sensitive phase contrast approach based on multi-distance image acquisition, which is able to retrieve the phase shifts from different images (at the expense of a higher radiation dose) using specific image processing algorithms [20].

Imaging was conducted at the European Synchrotron (ESRF, France) and the Swiss Light Source (SLS, Switzerland).

### Micro and sub-micron X-PCI-CT

The micro X-PCI-CT experiment was conducted at the ID17 biomedical beamline (ESRF). The X-ray beam, delivered by a 21-pole wiggler, was monochromatized by a double [111] silicon crystal system in Laue geometry [23, 24] with an energy of 60 keV. The source-to-sample and sample-to-detector distances were 145 m and 11 m, respectively. As an imaging detector, the PCO.edge 5.5 sCMOS camera (Kelheim, Germany) was used with an effective pixel size of 6.1 × 6.1 µm^2^ [20, 25]. For CT imaging, 4000 projections of the samples over 180° were acquired with an exposure time of 25 ms per projection.

Sub-micron experiments were conducted at both the ID17 beamline (ESRF) and the TOMCAT beamline (SLS). The experiment at ID17 was performed with a filtered polychromatic beam using a peak energy of 55 keV and a sample-to-detector distance of 1.2 m. As a detector system, a PCO.edge 5.5 camera with a 10 × Optique Peter (Lentilly, France) was used, achieving an effective pixel size of 0.7 × 0.7 µm^2^ [26]. CT acquisitions consisted of 50 ms per projection.

For the sub-micron experiment at the TOMCAT beamline, a monochromatic X-ray beam, achieved using a double multilayer monochromator [27], with an energy of 17 keV was used. The sample-to-detector distance was 0.1 m using the PCO.edge 5.5 camera with a 20 × Optique Peter magnification detection system, thus providing an effective pixel size of 0.325 × 0.325 µm^2^; 2000 projections over 180° were acquired with an exposure time of 200 ms each.

All micro- and submicron experiments were single distance phase retrieval experiments with the sample stage in normal pressure.

### Nano-holotomography

The ID16A nano-imaging beamline (ESRF) has a dedicated instrument for X-ray nano-holotomography experiments where biological tissues can be analyzed with effective pixel sizes down to 0.025 × 0.025 µm^2^ [28]. The beamline is equipped with two undulators and is optimized for focusing hard X-ray beams 1% at energies of 17 keV and 33.6 keV. The energy used for our experiment was 17 keV. A Kirkpatrick–Baez pair of mirrors focuses the beam down to a spot of about 0.03 × 0.03 µm^2^ [29]. The sample stage is placed downstream of the focal spot and, given the beam divergence (geometrical magnification effect), different effective pixel sizes can be chosen by placing the sample at specific distances with respect to the beam focus and to the detector. For our measurements, an effective pixel size of 0.1 × 0.1 µm^2^ was set. The sample environment is under vacuum. For each sample, four CT scans at different sample-to-focus-to-detector distances were acquired. The alignment of projections, phase retrieval and reconstruction were processed using protocol as described in Pacureanu et al. 2018 [28].

### CT image reconstruction and post-processing

CT data were reconstructed using the filtered back-projection (FBP) algorithm [30, 31]. The algorithm is implemented in the PyHST2 package [32] including also routines for ring artifact correction and single-distance phase-retrieval. For post-processing, the software ImageJ [33] was used. A method called Z-projection was applied to project subvolumes of 3D data into a 2D image. In our case, we projected the minimum intensity value of 200 slices to produce a 2D image with all the minimum values along the sagittal axis of the cartilage (perpendicular to former joint surface). Chondron segmentation was realized using the WEKA machine learning pixel classification tool [34] and 3D rendering with the software VGStudio max [35].

### Histology

After imaging, the cartilage samples were embedded in paraffin and cut into 5 µm thick sections, obtained perpendicular to the former joint surface of the central cylinder. The sections were stained using hematoxylin/eosin (H&E) and Masson–Goldner trichrome. The H&E stains cell nuclei blue, cytoplasmatic proteins, mitochondria, smooth endoplasmic reticulum and collagen red and provides an overview of the tissue’s cellular components. The Masson–Goldner trichrome stains cell nuclei brown/black, cytoplasm red, collagen and proteoglycans green thus revealing collagen and proteoglycan content. The histological images were acquired with a Zeiss Axiophot microscope equipped with an Axiocam HRc camera using Plan-Neofluar × 5 and × 20 objectives (Zeiss Oberkochen, Germany).

### Transmission electron microscopy

The samples for transmission electron microscopy (TEM) were removed from the un-calcified cartilage region and post-fixed in Karnowski fixative, which was prepared as a mixture of 25 ml paraformaldehyde (8%) solution, 10 ml glutaraldehyde (25%) solution and 15 ml Sörensen buffer (0.1 M). All solutions were freshly prepared. The cartilage samples were then embedded in Araldite CY212 and cut at a thickness of 60–70 nm using a diamond knife. The TEM images were acquired with a Zeiss EM 900 model, equipped with a slow-scan CCD camera with a 2k-wide angle. TEM images were acquired with 50 kV, a magnification factor of respectively × 3000 and × 12,000, which corresponds to a pixel size of 1.2 × 1.2 nm^2^ and 0.3 × 0.3 nm^2^ respectively.

## Results

### Multiscale X-PCI-CT for hierarchical imaging of cartilage: Variation of spatial resolutions

**Fig. 1:**
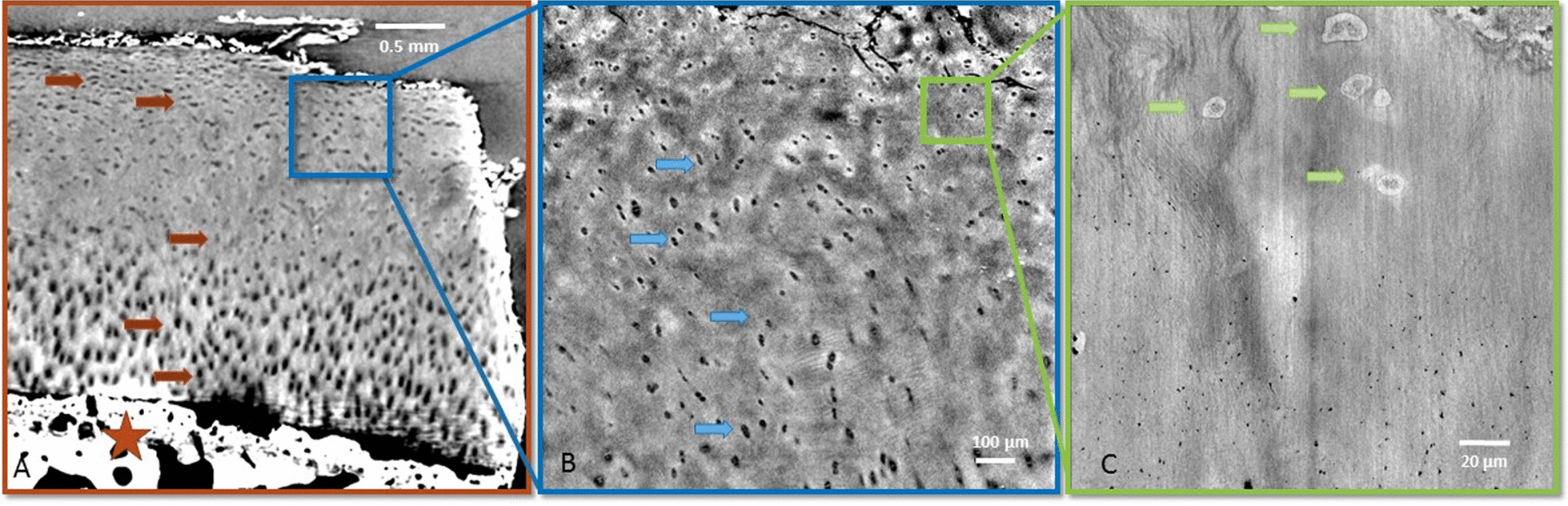
X-PCI-CT sagittal images of the healthy cartilage tissue acquired at different spatial resolutions. Arrows indicate chondrocytes. **A** X-PCI-CT image (6.1 × 6.1 × 6.1 µm3 voxel size, 60 keV X-rays, single distance) providing an overview of the cartilage tissue with its different layers: the star indicates the subchondral bone. **B** X-PCI-CT image (0.7 × 0.7 × 0.7 µm3 voxel size, filtered polychromatic X-ray beam, single distance) of a sub-region within the cartilage (blue rectangle in **A**), where numerous chondrocytes are visible. **C** X-PCI-CT image (0.1 × 0.1 × 0.1 µm3 voxel size, 17 keV X-rays, nano-holotomography) showing details of the cell structures within the chondrocytes and matrix components. In A + B lighter grey indicates higher densities while darker gray indicates lower tissue densities, whereas in **C** it is the opposite. Faint bright vertical strips visible in the nano-holotomography image (**C**) are artifacts from uneven surface edges caused by the cutting process during sample preparation

X-PCI-CT images of cartilage samples acquired at three different spatial resolutions (voxel sizes of 6.1 × 6.1 × 6.1 µm^3^, 0.7 × 0.7 × 0.7 µm^3^ and 0.1 × 0.1 × 0.1 µm^3^) provide a detailed depiction of cartilage structures at different magnifications and fields of view (inversely proportional to the magnification given the constant number of detector pixels). Depending on the chosen spatial resolution images encompassing the entire cartilage layer and the subchondral bone (overview frame) or individual magnified portions of cartilage matrix and its intracellular components (detailed frame) can be acquired for further analysis (Fig. 1).

The difference in greyscale on the X-PCI-CT images accounts for tissue density gradients, brighter areas indicating higher and darker areas lower tissue densities (Fig. 1).

### Overview of the cartilage architecture and chondrocyte arrangement

**Fig. 2:**
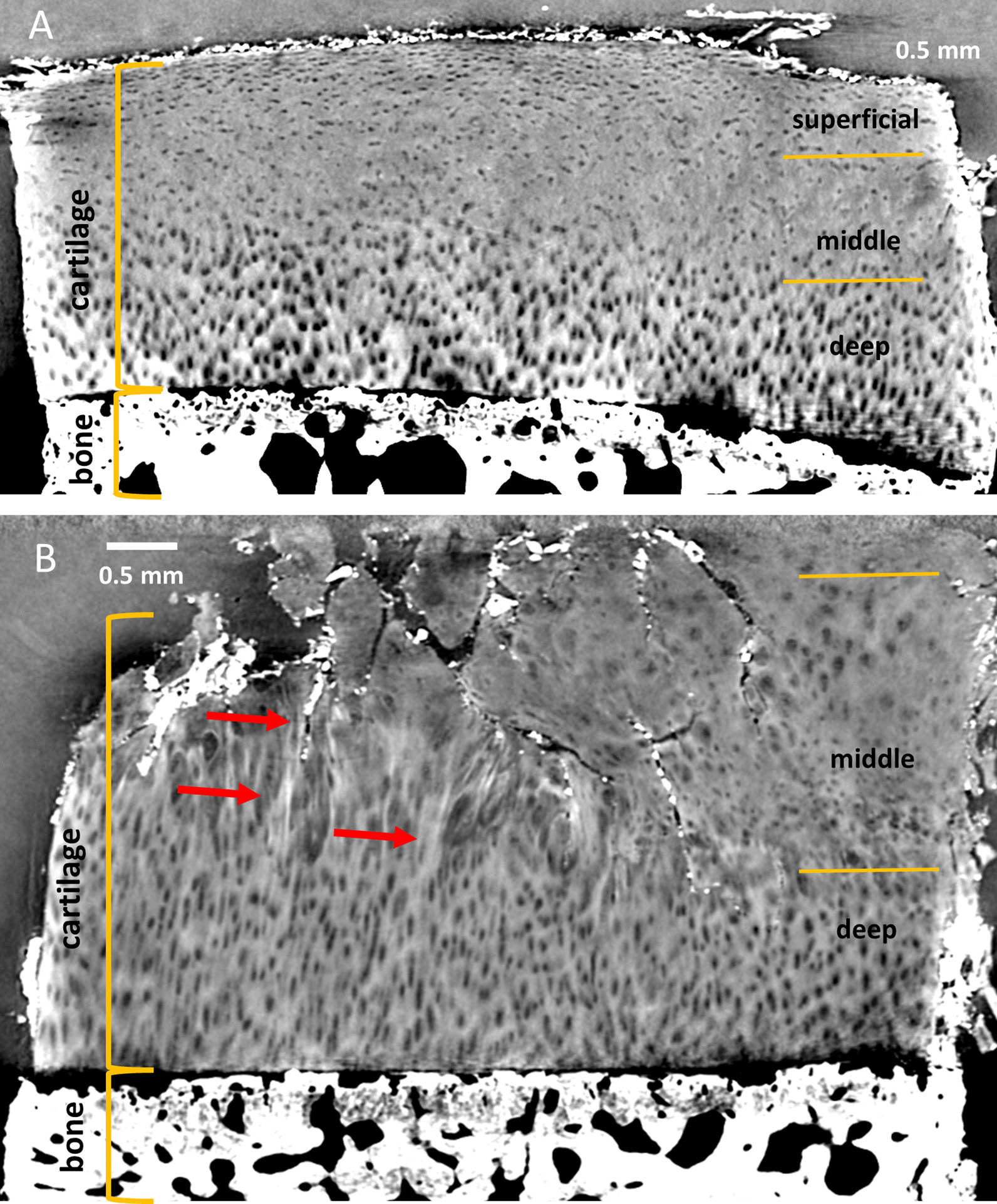
X-PCI-CT images of a healthy (**A**) and moderately degenerated (**B**) cartilage sample, respectively. Images were acquired using X-rays of 60 keV and a voxel size of 6.1 × 6.1 × 6.1 µm3 at the ID17 beamline. **A** The three cartilage layers are visible and intact (superficial, middle and deep layer); **B** The superficial layer and the middle layer show deep defects and fissures with disturbances of the matrix architecture (red arrows), while the deep layer is still preserved. The chondrocytes in the degenerated sample (**B**) show changes in their morphology throughout the superficial layer with loss of their flattened shape in the superficial layer as well as enlargement and initial clustering in the middle layer

**Fig. 3:**
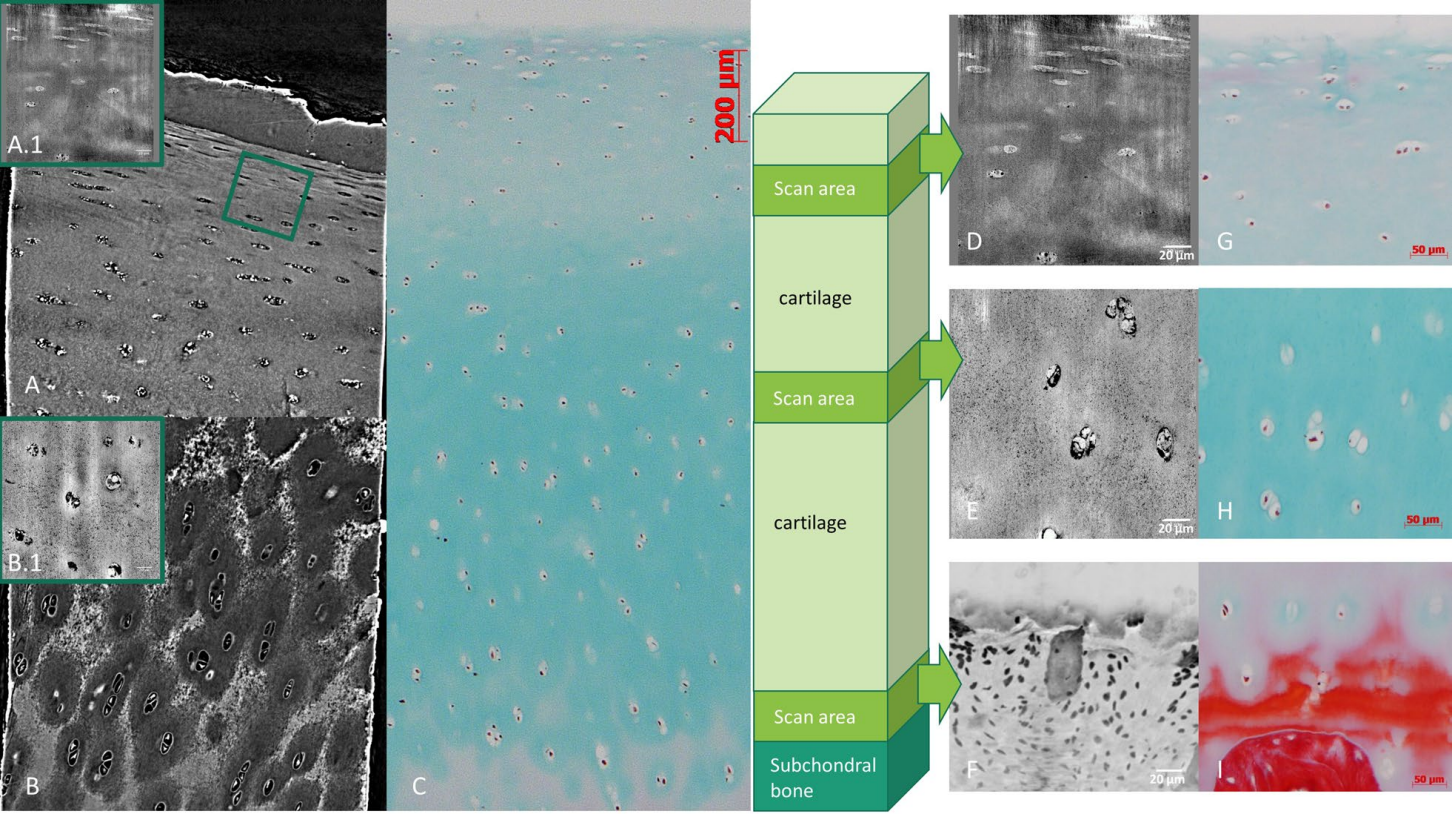
X-PCI-CT images of a healthy sample compared with corresponding histological images. **A** superficial layer and **B** middle/deep layer (TOMCAT beamline, voxel size 0.325 × 0.325 × 0.352 µm3); **A.1** + **B.1** + **D** + **E** nano-holotomography images from ID16A (0.1 × 0.1 × 0.1 µm3 voxel size); **F** Z-projection X-PCI-CT image of the tidemark (ID17, ESRF, 0.7 × 0.7 × 0.7 µm3 voxel size); **C** Histological images (scale bar 200 µm) of superficial, middle and deep layers (Masson Goldner trichrome staining: cell nuclei brown/black, cytoplasm red, collagen and proteoglycans green); **G**-**I** Histological images (scale bar 50 µm) of superficial, middle and deep layers (Masson Goldner trichrome staining)

Sagittal X-PCI-CT data reconstructions acquired with a voxel size of 6.1 × 6.1 × 6.1 µm^3^ provide an overview of the tissue architecture by depicting the complete cartilage layer with its characteristic three layer composition (superficial, middle, deep layer). X-PCI data confirm varying cell arrangements throughout the tissue depth (Fig. 2 healthy (A), degenerated (B) cartilage sample) with differing cell sizes, distribution and orientations. These visible cells correspond to individual chondrocytes and groups of chondrocytes (chondrons) as shown in consecutive histology (Fig. 3C).

Tissue degradation signs, such as superficial defects, their in-depth spread, but also concomitant tissue architecture alterations with disturbances of the matrix, chondrocyte arrangement and even changes in morphology are directly visualized (Fig. 2B).

### Detailed sub-cellular analysis within healthy cartilage matrix shows cartilage shape and content with good anatomical correlation to histology and TEM

The sub-micron resolution X-PCI-CT datasets from the TOMCAT beamline (0.325 × 0.325 × 0.325 µm^3^ voxel size), ID16A (0.1 × 0.1 × 0.1 µm^3^ voxel size) and ID17 (0.7 × 0.7 × 0.7 µm^3^ voxel size) visualize chondrocytes and tissue morphology at a higher level of detail as compared to CT and MRI and correlated well with histological slices obtained with Masson–Goldner trichrome staining (Fig. 3). Within the chondrocytes, a round core is resolved, which represents the cell nucleus. Around the cell nucleus and within the cell boundary multiple coarse dark depositions are visualized, which might represent either small cell components or artifacts.

In the superficial, also thinnest, layer of the cartilage matrix, the chondrocytes possess a flattened shape with horizontal orientation (long axis parallel to tissue surface, Fig. 3A + D). In the middle layer, the chondrocytes reveal a round to oval shape and are arranged in pairs, triples or small clusters without a special arrangement (Fig. 3E). In the depth of the cartilage, the third, thick layer of the tissue shows round to oval-shaped chondrocytes, arranged in long columns with cells oriented perpendicularly to the subchondral bone surface (Fig. 3B). At the cartilage-bone interface, the chondrocytes form cluster again, partially within the deepest layer of the mineralized cartilage. The transition zone between cartilage and mineralized cartilage, the so-called tidemark, is well visible and shows a wavy interface (Fig. 3F). Below that, the mineralized cartilage and the subchondral bone are separated by a cementline and the bone architecture is depicted with cell bodies arranged around lacunar structures (Fig. 3F).

These images reveal a tissue gradient within the cartilage matrix from the superficial to the deep layer (Fig. 3A + B) as well as denser matrix variations around the cell cluster in the middle/deep layer (Fig. 3B), which correspond to a color-gradient seen in the Masson-Goldner-staining (Fig. 3C) suggesting corresponding variations in the collagen-proteoglycan content.

### Detailed sub-cellular analysis within mildly degenerated cartilage matrix shows architectural disruption and detailed chondrocyte cell shape transformation previously not visible with X-ray imaging

**Fig. 4:**
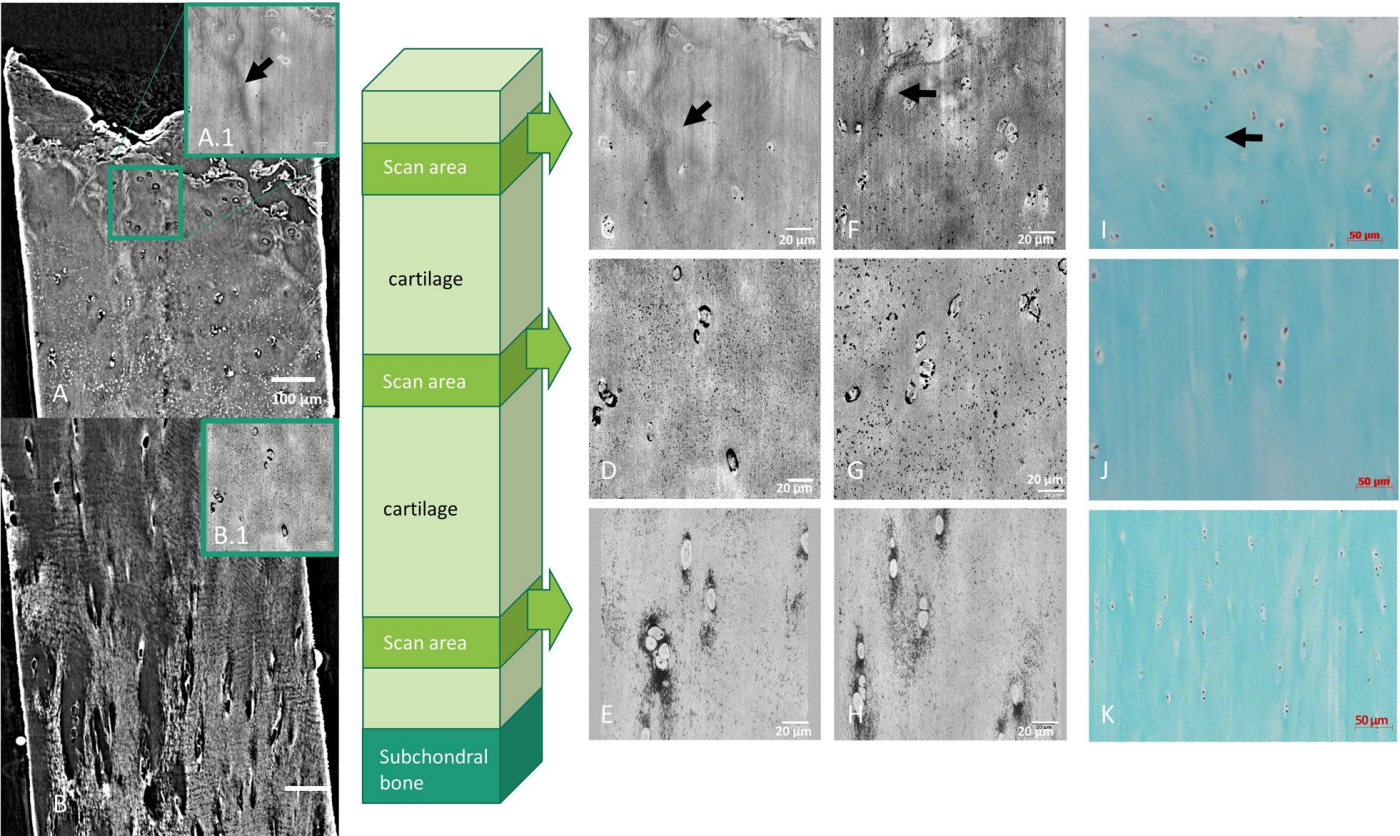
X-PCI-CT and histological images of a mildly degenerated cartilage sample from superficial, middle and deep layers. **A** + **B** CT images from TOMCAT beamline (0.325 × 0.325 × 0.325 µm3 voxel size); **A.1** + **B.1** + **C**-**H** nano-holotomography images from ID16A (0.1 × 0.1 × 0.1 µm3 voxel size; **C** + **F** in these nano-holotomography images of the superficial layer fiber bundles are visible (black arrow); **D** + **G** nano-holotomography images of the middle layer with cell clusters; **E** + **H** nano-holotomography images of the deep layer, vertical clustering of cells; **I**–**K** Histological images of the corresponding cartilage layers of the same row (scale bars 50 µm, Masson Goldner trichrome staining)

**Fig. 5:**
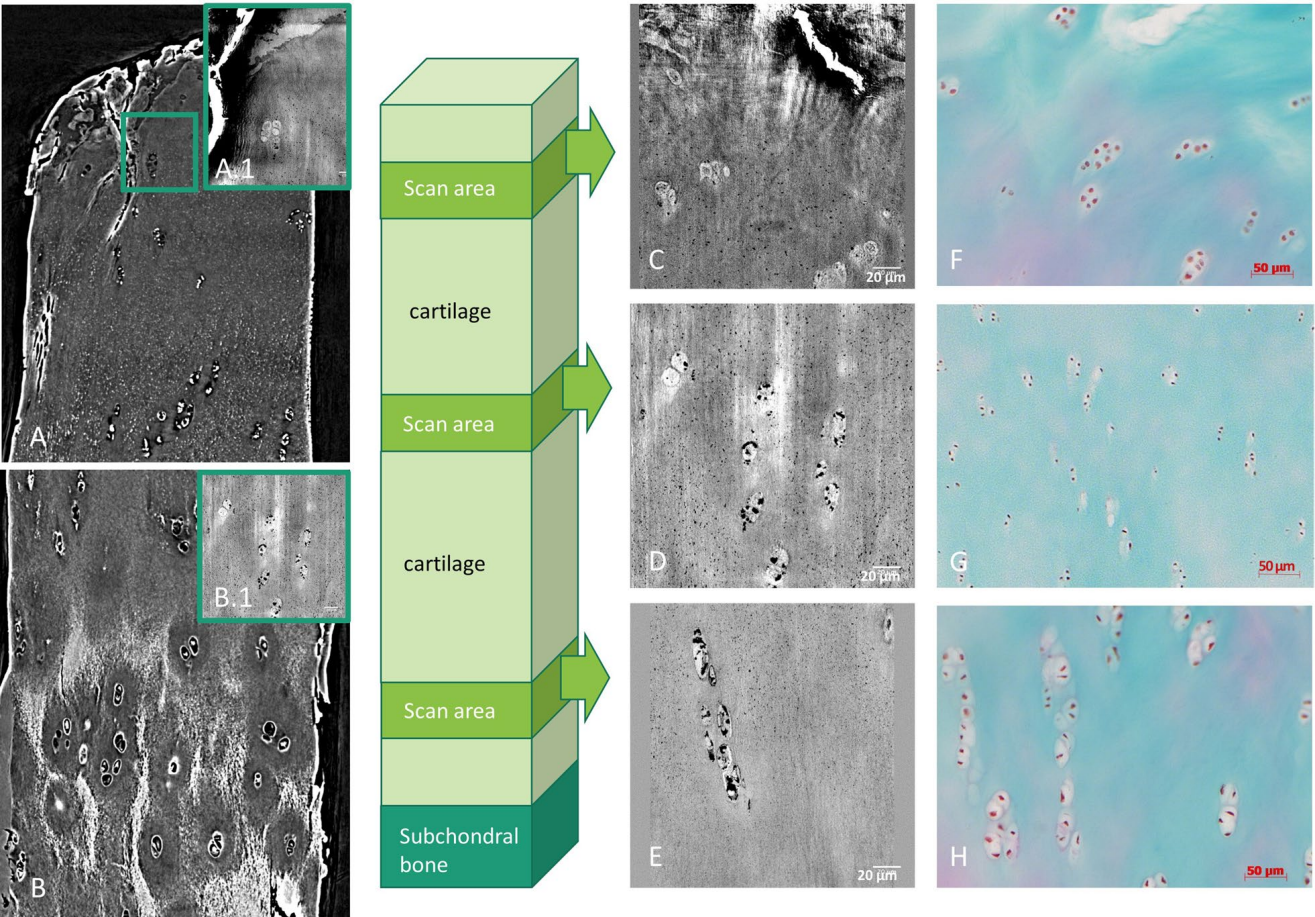
X-PCI-CT images compared to histological images of a degenerated cartilage sample. **A** + **B** X-PCI-CT images from TOMCAT beamline (0.325 × 0.325 × 0.325 µm3 voxel size); **C** + **D** + **E** nano-holotomography images from ID16A (0.1 × 0.1 × 0.1 µm3 voxel size); **A.1** + **C** superficial layer with cracks; **D** middle layer cells clustering; **E** deep layer cells clustering in vertical direction; **F** + **G** + **H** Histological images from superficial, middle and deep layers (scale bars 50 µm, Masson Goldner trichrome staining)

The X-PCI-CT images acquired with a 0.1 × 0.1 × 0.1 µm^3^ voxel size of the mildly degenerated samples reveal different degrees of fibrillation, tears, cracks and/or delamination of the superficial and often of the middle layers as direct signs of damage (Figs. 4, 5).

Besides surface defects, changes in morphology and chondrocyte arrangement are observable (Fig. 4C + F). In the disrupted and partially dissipated superficial layer, the former flattened chondrocytes are rarefied and enlarged, their shape in and just below that layer morphed into rounded and their originally ordered orientation broken into a more scattered formation (Figs. 4C, 5C).

The former rounded middle layer chondrocytes appear enlarged and form clusters of differently sized cells (Figs. 4D + G, 5D).

In the deep layer, the chondrocytes are still arranged in columns, but a slight disarrangement is seen due to partial clustering (Figs. 4E + H, 5E).

### High-resolution details of intracellular cartilage anatomy and pericellular environment

The cell bodies show varying regional intracellular densities and a halo around single and clustered cells (Figs. 4B, 5B). Several samples present numerous high-density dots (diameters between 1 and 10 µm) within the chondrocytes and at the proximity of their border, accentuated in the middle and deep cartilage layers (Figs. 3E, 4D + E, 5D–E). Many samples also show tiny disseminated black spots within the whole matrix (e.g. Figs. 3E, 4C–H, 5C–E) albeit the health status. In one sample, fiber-like linear dense structures are visible in between the chondrocytes in the superficial layer (Fig. 4A + C + F).

### Possible post-processing techniques to improve cartilage matrix analysis

**Fig. 6:**
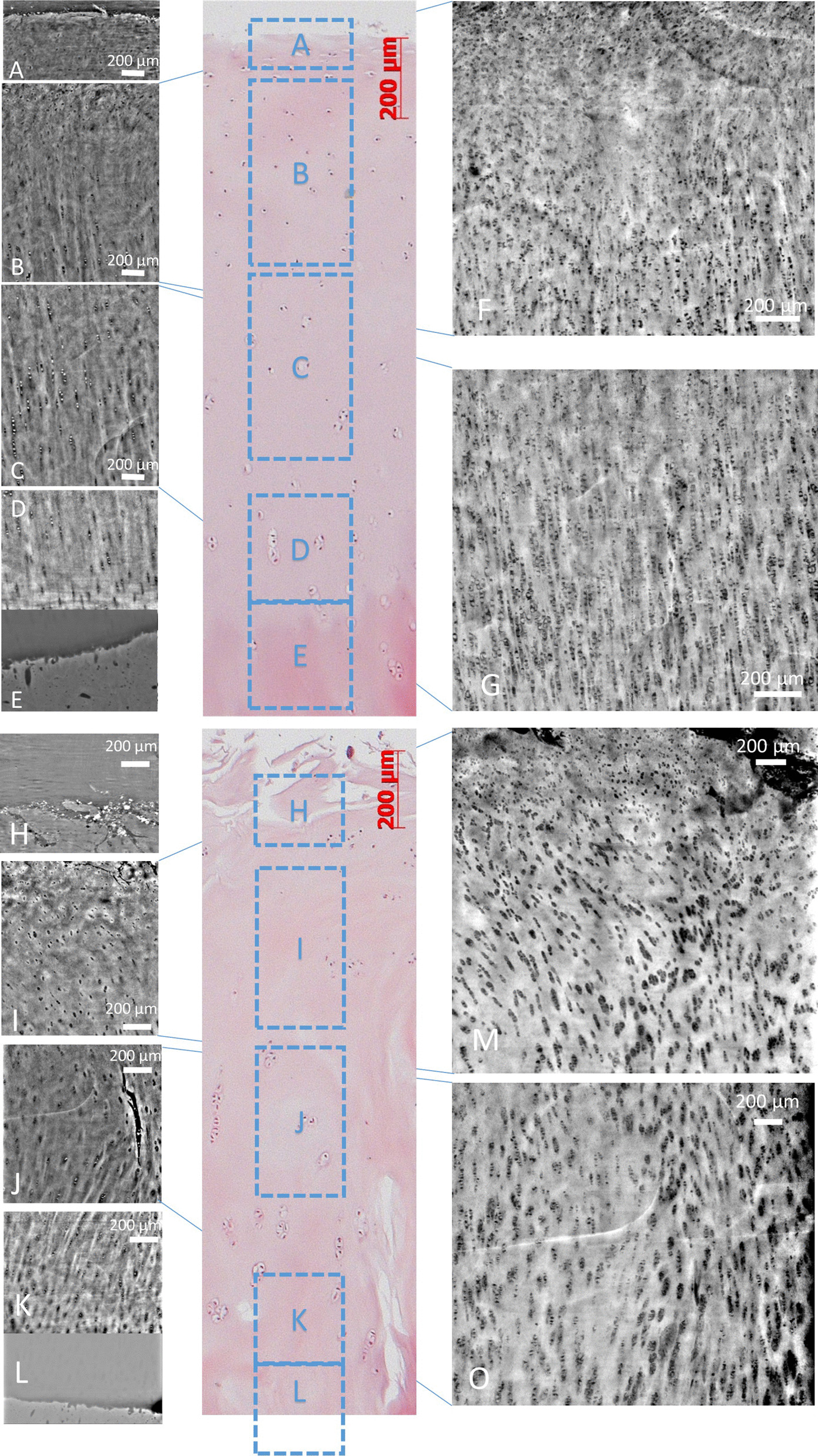
X-PCI-CT images of healthy sample **A**-**G** (sagittal reformation) and degenerated sample **H**–**O**; images acquired at the ID17 beamline with an effective voxel size of 0.7 × 0.7 × 0.7 µm3. In the center column of the panel: a histological H&E stained slice. **F** + **G** + **M** + **O** Layered X-PCI-CT images (i.e. Z-projections) from the upper and lower middle layers (minimum pixel values layered of 200 slices). **A** superficial layer; **B** part of the superficial and middle layers; **C** part of the middle and deep layers; **D** deep layer; **E** deep layer with adjusted gray level for tidemark visualization; **F** chondrocytes are partially aligned in an arc to support the superficial layer; **G** chondrocytes are oriented vertically in the transition zone between middle and deep layer along the fiber orientation. **H** superficial layer with uneven surface; **I** middle layer; **J** middle layer with a fissure; **K** deep layer; **L** deep layer with adjusted gray level for tidemark visualization; **M** superficial layer with rough surface area, chondrocytes are aligned in arc to support the existing superficial layer; **G** chondrocytes are oriented vertically in the transition zone between middle and deep layer along the fiber orientation

Image post-processing allows for layering of multiple X-PCI-CT images, which increases the number of depicted structures in one image and provide a slight 3D aspect to the slice. This data representation improves the revelation of localization and arrangement of visible tissue components and therefore overall cartilage architecture with its arcade-like chondrocyte distribution in the middle layer (Fig. 6B + F) below the superficial layer (Fig. 6A) and the column-like arrangement of the chondrocytes in the deep layer in a healthy sample (Fig. 6C + D + G + K + O). The tidemark is also clearly visible as a wavy interdigitating surface in the horizontal plane (Fig. 6E + L) between cartilage and subchondral bone.

Given the larger number of depicted chondrocytes in the layered images, the pattern of disruption of the chondrocyte arrangement in the degenerated samples appears more clearly (Fig. 6H-J + M).

### 3D chondrocyte distribution analysis

**Fig. 7:**
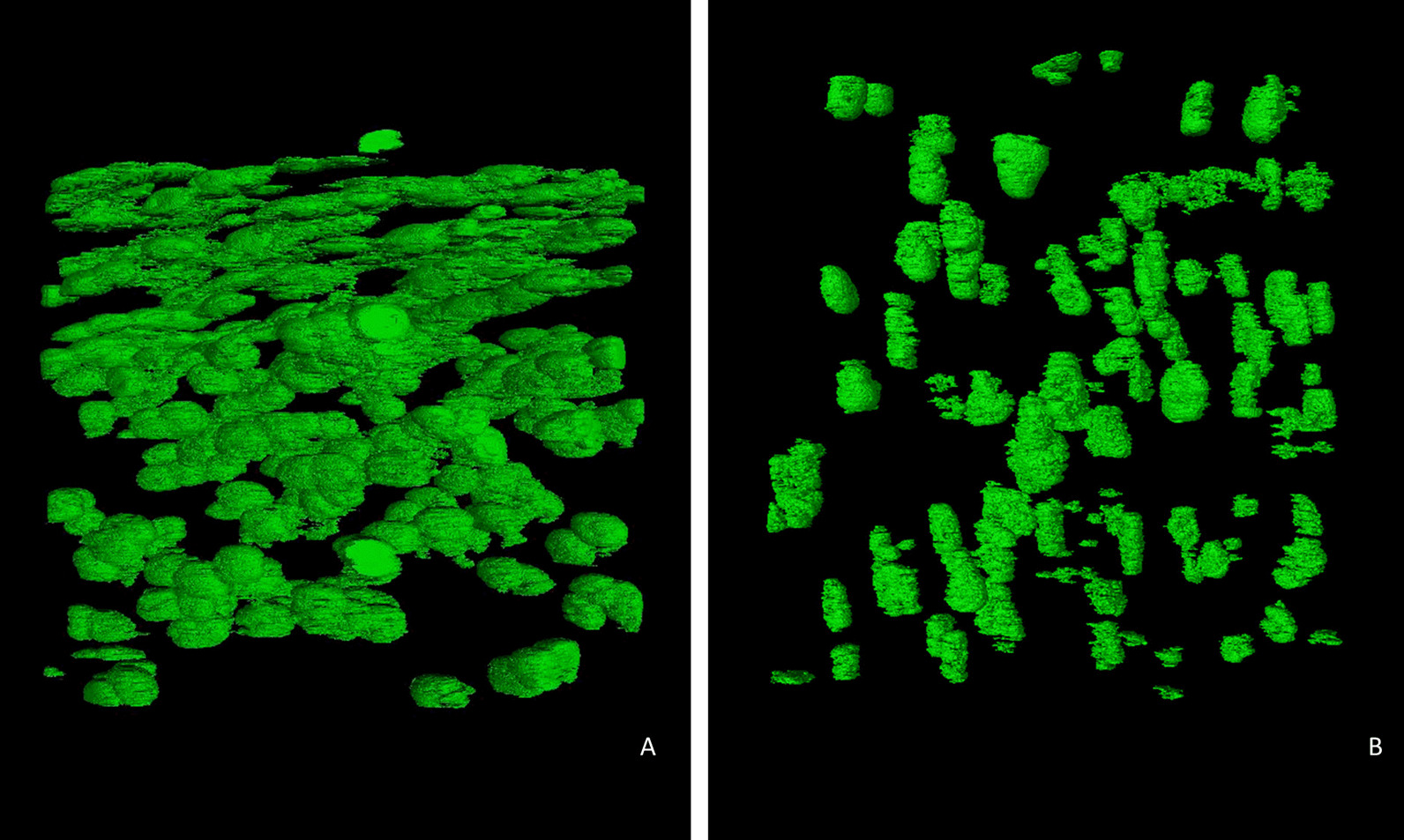
3D rendering (VGStudio max) of segmented chondrocytes/chondrons in a healthy cartilage sample (**A**) and a degenerated sample (**B**) in the superficial and underlying middle layers. The segmentation was performed with the advanced machine learning algorithm WEKA

3D rendering and segmentation of chondrocytes and chondrons in the nano-holotomography datasets (effective voxel size 0.1 × 0.1 × 0.1 µm^3^) were performed using the WEKA machine learning program [34] and results enable, aside from visualization, additional quantitative analysis of cell morphology, like shape, size and distribution within the matrix (Fig. 7). In the healthy sample (Fig. 7A) the superficial layer was preserved, recognizable by the layer of horizontally oriented flat cells. In the degenerated sample (Fig. 7B), the superficial layer was absent with morphed, more vertically oriented cell clusters. The 3D model clearly highlights how the different cartilage layers are mildly intertwined throughout the tissue.

### Nano-holotomography versus TEM

**Fig. 8:**
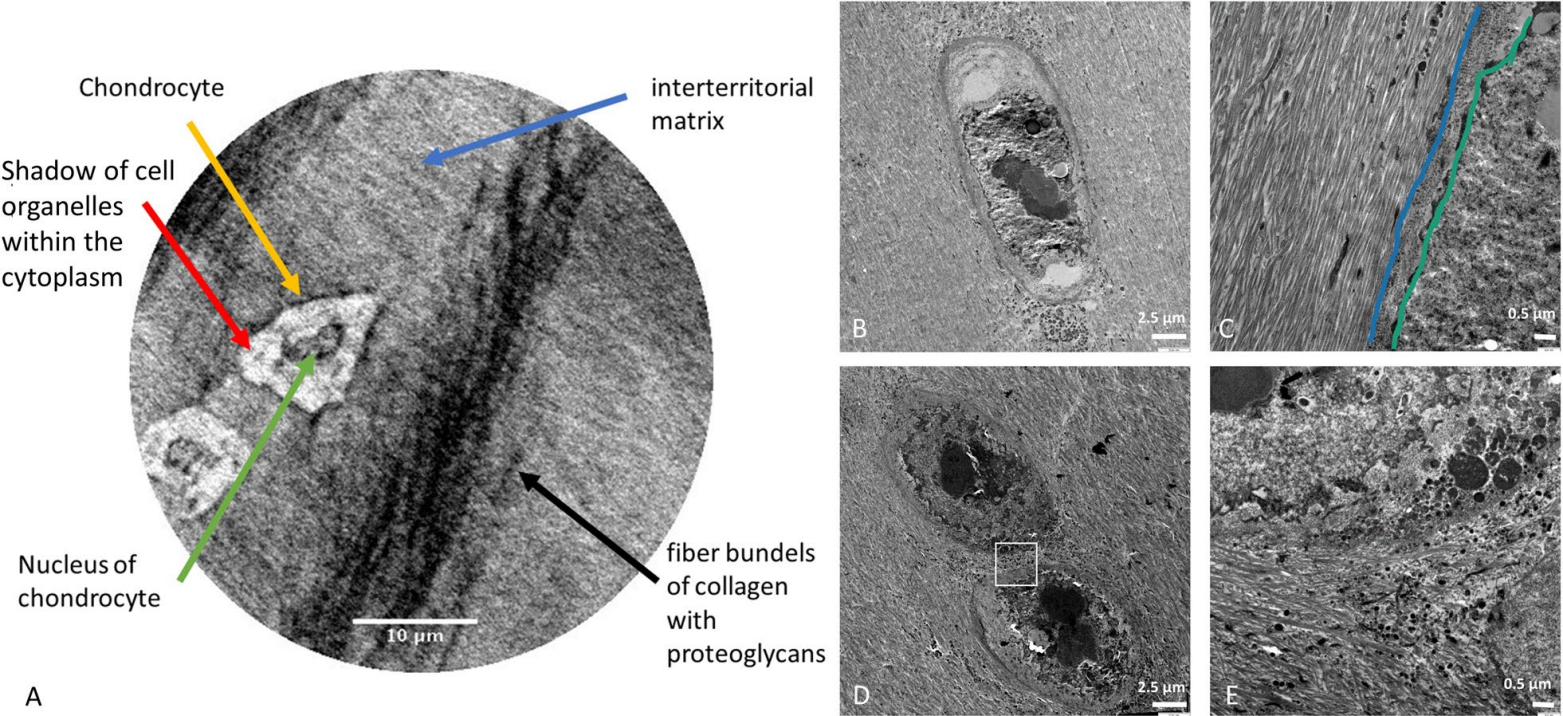
**A** Excerpt of a mildly degenerated cartilage sample from a nano-holotomography image (0.1 × 0.1 × 0.1 µm3 voxel size) showing two chondrocytes with their nucleus, shadows of cell organelles within their cytoplasm and unmasked fiber bundles, in between interterritorial matrix (scale 10 µm); **B**–**E** Transmission electron microscope images of cartilage; **B** + **D** chondron with × 3000 magnification (scale 2.5 µm); **C** Territorial matrix (left of blue line), immediately pericellular matrix (between lines), chondrocyte (on the right of the green line), × 12,000 magnification (scale 0.5 µm); **E** territorial matrix close up from image **D** (scale 0.5 µm)

X-ray nano-holotomography, the spatially highest resolved technique (0.1 × 0.1x0.1 µm^3^), depicts single cells and details within the cell body including the cell nucleus and even shadows of cell organelles approaching the shapes depicted by the even higher resolved TEM (Fig. 8A versus B + D). Similarly, variations of the surrounding territorial matrix around chondrocytes is observable, though still with less detail than in TEM (Fig. 8A versus C + E). In one mildly degenerated sample, bundle-like structures are unmasked within the tissue (Fig. 8A).

## Discussion

X-PCI-CT is the first, non-invasive, 3D imaging technique reaching the necessary resolution to visualize cellular and extracellular variation in cartilage matrix approximating histology and TEM analyses. The application of different spatial resolutions provides insights at different length scales into the tissue organization and can therefore serve as a complimentary laboratory based method for fundamental cartilage research to study detailed anatomical changes and their role in underlying mechanisms of OA evolution.

The multi-scale X-PCI-CT imaging approach provides a unique potential for volumetric and highly detailed cartilage examination. Images with a high degree of detail can be obtained not only for single, sparse slices, but also for the full volume of the examined tissue allowing for the analysis of the specimen in its entireness. It is possible to visualize its defined intertwined three-layered organization [36] as well as the wavy construction of the tidemark, which together with the intertwined layers might be of relevance for force distribution and biomechanical stability. This kind of information could not be extracted in the similarly highly resolved histology or TEM images. Having a volumetric dataset additionally provides the possibility of selecting and viewing arbitrary planes within the reconstructed volume, enabling the researcher to navigate virtually through the tissue volume and improve correlation of micro and sub-micro X-PCI findings in the examined tissue with current validated techniques such as histology and TEM. X-PCI therefore adds what might be called “X-ray virtual 3D histology” capturing tissue within their native compound gaining information with respect to radiodensity on a 3D level, which might give more insight about cartilage tissue properties as compared to conventional 2D histology or TEM on a comparable scale.

In addition, the sample preparation for X-PCI measurements is rather simple if compared to that required for histology and TEM. Basically, samples only need fixation in formalin or in equivalent agents; for nano-imaging acquisition embedding in paraffin is helpful to avoid sample degradation due to radiation doses. Expect for these few steps, no staining nor further preparation is required and therefore tissues are imaged in a condition closer to their natural state. Histology, instead, needs multiple preparation steps such as decalcification, conservation, cutting (with the risk of destroying the sample) and staining to produce histological images.

High-resolution imaging of human cartilage with X-PCI is reported in recent literature to reach a micron resolution [37, 38], which only depicts the cell distribution. Therefore, another innovation is that nano-tomography achieves a sub-micron resolution, which enables depiction of details within a single cell and for the first time as a radiographic technique the depiction of tissue density variations around chondrocytes corresponding to a differing biochemical composition (e.g. collagen, proteoglycan, other biochemical molecules) of the extracellular matrix, which is reportedly produced and altered by chondrocytes [39]. The varying chondrocyte shapes from flat superficially [40], round in the middle [41] and larger rounded in the deep layer [42] endorse the theory that morphology might indicate a different function for matrix homeostasis and that their change might induce disruption of this homeostasis.

Fiber bundle-like structures, probably representing different collagen fiber types, were depicted in one sample and only in the nano-holotomography data; it remains therefore unclear under what experimental conditions collagen might be visible. As this occurred in one mildly degenerated sample, a possible explanation is the unmasking of collagen bundles due to loss of proteoglycans, a phenomenon known from histology, but not yet examined in X-ray imaging.

There are two types of sediments, whose nature remain unclear. Some cartilage samples show disseminated tiny dark spots, which may represent residual sediments artificially caused by formalin storage time or tissue preparation. A second coarser type accumulated around the chondrocyte cell border, which, given the location, might correspond to small cell components, but also artifacts regarding missing equivalents in histology. These results suggests that X-PCI provide complementary imaging information to conventional laboratory imaging techniques, which however need further research to evaluate their significance.

Overall, X-PCI in combination with ad-hoc 3D image processing and rendering tools could serve for generating a pictorial reference atlas, capturing the stages of the aging process, and help consistently classifying cartilage lesions, beneficial for training, comparison and research purposes. Identifying the sequence of cartilage degradation might aid in understanding functional tissue behavior and the nature of tissue degradation and healing, which is crucial for identifying potential points for therapeutic interventions such as stopping, delaying or future replacement of cartilage defects with functional tissue.

### High-resolution X-PCI-CT with synchrotron radiation

The ID17 beamline (ESRF) and the TOMCAT beamline (SLS) are equipped with multi-scale, high sensitivity X-PCI-CT setups that enable scan times of ~ 10 min per CT scan on a sample with dimensions of a few millimeters-centimeters. Sample environment is under atmospheric pressure and the sample preparation only require conservation. The TOMCAT beamline reaches pixel sizes down to 0.16 × 0.16 µm^2^, while the smallest pixel size achievable at ID17 is 0.7 × 0.7 µm^2^. ID16A (ESRF) provides the highest spatial resolution (0.1 × 0.1 µm^2^ effective pixel size) with an acquisition time of about 4 h in total, since 4 CT scans (4 × 1 h; i.e. at 4 sample-to-detector distances) are required for a complete nano-holotomography examination per sample, which for this experiment are kept within a chamber under vacuum.

The ID17 and the TOMCAT beamlines provide fast acquisition and uncomplicated sample preparation in comparison to the ID16A beamline. But, the ID16A beamline provides the highest resolution and a very accurate, quantitative phase information about the cartilage due to the nano-holotomography technique. The combination of multiple CT scans exposes the samples to a higher radiation dose, but signs of radiation damage were neither observed in the macroscopic or histologic sample nor in the acquired data. Therefore, the novel sample preparation for the high-resolution imaging under vacuum was successful.

### Limitations of the study

Synchrotron radiation facilities provide the highest image quality and the shortest acquisition times for X-PCI-CT applications because of their high brilliance and coherence properties compared to laboratory X-ray tube sources, but are not easily accessible. Today X-PCI-CT can be applied in laboratories using novel, advanced X-ray tube technologies with more compact one-room X-ray light sources for X-PCI-CT applications [43–46], which permit this technique to applied outside the synchrotron setting for specific applications in particular for those where the requirements in terms of spatial resolution and acquisition times are not very stringent. These technologies can fit a large room within a hospital or a university department similarly to today’s CT or MRI devices. Technically X-PCI is a tissue-conserving imaging technique, and even though larger objects are penetrable and depictable [17, 47], the micrometer cellular resolution is still reachable only in small samples, because of the current technological limitations in having large field-of-view detectors. Thus, tissue samples still need to be harvested and reduced in size, which limits the analyzable area substantially. In addition, high spatial resolution imaging, while providing a high level of information on the studied specimen, needs specific, sufficiently high photon fluxes in order to limit the acquisition time. This results in a trade-off between spatial resolution and radiation dose, which for now limits the possibilities for sub-micrometer imaging investigations for in-vivo and clinical human imaging. Therefore, sub-micron scale imaging today is only possible in an ex-vivo/post-mortem laboratory setting.

Given the technical requirements, our examinations were limited to only 10 different human cartilage samples. Yet, the applied protocol allowed visualization of 3D architecture in samples at a scale previously unreachable with other destruction-free methods in cartilage.

## Conclusions

This study proves the potential of multi-scale X-PCI-CT for investigating cartilage architecture and composition on a cellular and subcellular level using micron and sub-micron resolution with a 3D volumetric approach and good correlation to respective changes in histological and TEM images. Knowing that chondrocyte subpopulations influence matrix homeostasis and possess differing colony-forming capabilities, techniques allowing to study morphological chondrocyte changes and migration might aid in understanding matrix composition, architectural alterations and biomechanical properties throughout the complex development of cartilage degradation. Therefore, X-PCI is a valuable candidate for complimentary non-invasive imaging of subtle morphological changes in early stages of cartilage alteration in a basic research/laboratory setting, which is important for the development of therapeutic options. The technique needs refinement for future tissue-conserving applications on larger objects; presently the main limitations are the high fluxes and the duration of a single examination when sub-micrometer voxel sizes are used. These factors and the associated high radiation dose level (from hundreds to thousands of milli-Gray for sub-micrometer investigations) limits the use of X-PCI-CT for in-vivo investigations at high spatial resolutions like those used in this study. However, this methodology already represents a valuable tool for research in ex-vivo/in-vitro human or animal samples, where radiation-induced degradation of biological tissues has never been detected.

## Data Availability

The datasets generated and/or analysed during the current study are not publicly available due their large size, but are available from the corresponding author on reasonable request.

## Abbreviations

2D: Two-dimensional
3D: Three-dimensional
CCD: Charged-coupled device
CT: Computed tomography
ESRF: European synchrotron radiation facility
FBP: Filtered back-projection
H&E: Hematoxylin and eosin
MRI: Magnetic resonance imaging
OA: Osteoarthritis
SLS: Swiss light source
TEM: Transmission electron microscopy
X-PCI: X-ray phase-contrast imaging
yo: Year old
